# Light-Controlled Swarming and Assembly of Colloidal Particles

**DOI:** 10.3390/mi9020088

**Published:** 2018-02-19

**Authors:** Jianhua Zhang, Jingjing Guo, Fangzhi Mou, Jianguo Guan

**Affiliations:** State Key Laboratory of Advanced Technology for Materials Synthesis and Processing, International School of Materials Science and Engineering, Wuhan University of Technology, Wuhan 430070, China; zhangjianhua4987@whut.edu.cn (J.Z.); october@whut.edu.cn (J.G.)

**Keywords:** light control, colloidal particles, swarm, assembly, collective behaviors

## Abstract

Swarms and assemblies are ubiquitous in nature and they can perform complex collective behaviors and cooperative functions that they cannot accomplish individually. In response to light, some colloidal particles (CPs), including light active and passive CPs, can mimic their counterparts in nature and organize into complex structures that exhibit collective functions with remote controllability and high temporospatial precision. In this review, we firstly analyze the structural characteristics of swarms and assemblies of CPs and point out that light-controlled swarming and assembly of CPs are generally achieved by constructing light-responsive interactions between CPs. Then, we summarize in detail the recent advances in light-controlled swarming and assembly of CPs based on the interactions arisen from optical forces, photochemical reactions, photothermal effects, and photoisomerizations, as well as their potential applications. In the end, we also envision some challenges and future prospects of light-controlled swarming and assembly of CPs. With the increasing innovations in mechanisms and control strategies with easy operation, low cost, and arbitrary applicability, light-controlled swarming and assembly of CPs may be employed to manufacture programmable materials and reconfigurable robots for cooperative grasping, collective cargo transportation, and micro- and nanoengineering.

## 1. Introduction

**Figure 1:**
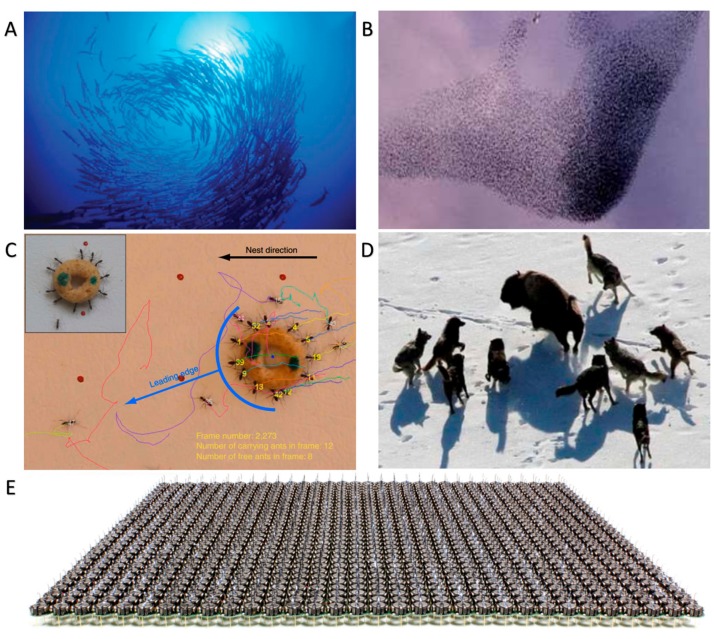
Biological and nonbiological swarms and assemblies. (**A**) A school of fishes (reproduced from [9]); (**B**) a flock of wild geese migrating (reproduced from [10]); (**C**) a group of ants carrying food (reproduced from [11]); (**D**) a pack of wolfs hunting a bison (reproduced from [12]); (**E**) a 2^10^-robot swarm (reproduced from [13], reprinted with permission from AAAS).

Swarming and assembly represent a process in which multiple entities aggregate together and/or organize into ordered or functional structures through interactions with each other and their environment [1,2,3,4,5]. Swarming and assembly are common phenomena in nature. The examples range from the stacking of atoms and pairing of DNA strands, to the formation of bacterial colonies, schooling of fishes, human crowds, and galaxies [6,7]. Unlike single individuals, the swarms and assemblies in nature may perform complex collective behaviors and cooperative functions. For instance, atoms with different stacking sequences create materials with different properties [8]. To avoid predators, increase their success rate of foraging, and adapt to environmental changes, many animals prefer to face these challenges in the form of swarms and assemblies. As shown in Figure 1A, large schools of fishes change their shape and internal structure to adapt to the surrounding environment [9]. Flocks of wild geese fly in the V formation to decrease the air resistance for migrating, and ant army can transport much larger and heavier food than their own bodies (Figure 1B,C) [10,11]. Similarly, wolves usually act in packs to perform many strategies for hunting and use the group power to defend themselves (Figure 1D) [12]. Some living organisms can communicate with each other by specific modes, like secreting chemicals and making sound waves, to aggregate, but in many cases they form aggregates spontaneously without a centralized control. Drawing inspiration from nature, a thousand-robot swarm could implement a programmable self-assembly [13]. Figure 1E demonstrates that these robots are located in a precise mode to collect information from their neighbors and fulfil a task cooperatively. The more robots there are, the more difficult the tasks that can be accomplished. Although some individuals inevitably commit errors, they can recover from the feedbacks sent by their neighbors when there is a large population of individuals. Therefore, swarms and assemblies represent a living way for biological individuals to face the challenges of a severe living environment and they are the guarantee for robots to accomplish complex and cooperative tasks.

Colloidal particles (CPs), including active and passive CPs, can serve as constituents to form swarms and assemblies. Active CPs, also defined as self-propelled particles or micro- and nanomotors, are CPs that can autonomously move by converting surrounding energies into their own kinetic energies [14,15,16,17], while passive CPs, differentiated from active CPs, are those that can migrate only under external forces. Both active and passive CPs are covered in this review. By mimicking their counterparts in nature, such as atoms, molecules, cells, and animals, they can be organized into complex structures in a controlled manner and exhibit cooperative functions [1,18,19,20,21,22,23,24,25,26]. The equilibrium assembly of CPs, which evolves the building blocks into stable, ordered structures as the system approaches equilibrium, has been studied for decades [27]. The non-equilibrium (or dynamic) swarming and assembly of CPs is promising to create biomimetic, reconfigurable, and ‘‘intelligent’’ materials, which are able to reversibly transform, disassemble, and even move in response to external stimuli [4,23]. Recently, a variety of strategies have been developed to realize the dynamic swarming and assembly of CPs by using different stimuli, such as chemical gradients and external fields (light, magnetic, electric, and ultrasound fields). Chemical gradients can trigger the motions and interparticle interactions of CPs by diffusiophoresis, leading to their swarming and assembly. For example, the spontaneous schooling of AgCl, Ag_3_PO_4_, TiO_2_–SiO_2_, and gold (Au) micro- and nanoparticles was demonstrated in the self-generated chemical gradient fields [28,29,30,31]. With a chemical gradient produced by a bone crack, negatively charged quantum dots, enzymes, and drug capsules can be dragged to the bone crack for bone crack detection, targeting, and repair [32]. Alternating current electric fields can induce aggregations of CPs with various emergent patterns, such as chains, swarms, and clusters, depending on the electric field frequency [33,34,35]. Similar to the electric field, the application of a rotating magnetic field (*H*) can maneuver the aggregation of superparamagnetic particles by magnetic dipole–dipole interactions [36]. For instance, Yan et al. reported that magnetic CPs tended to form linear chains under a low-strength rotating *H*, and then these chains dissolved and assembled into swarms once the *H* frequency reached 20 Hz. In addition, ultrasounds can create many pressure nodes, propelling CPs to move together following pressure gradients [37,38,39]. Light can trigger photoinduced interactions between CPs to form swarms and assemblies. For instance, autonomous TiO_2_–Pt micromotors under ultraviolet (UV) irradiation can effectively capture and assemble CPs into dynamic colloidal molecules based on light-controlled electrostatic interactions [40]. On the other hand, CP swarms can perform ample collective behaviors by adjusting to external stimuli. For instance, Ag_3_PO_4_ microparticle swarms exhibit expansion and contraction with the addition and removal of NH_3_, Pt–Au nanowire motor swarms exhibit overall migration under the control of ultrasounds, and the superparamagnetic particle swarms can perform rolling with the cooperative control of ultrasounds and magnetic fields [30,36,41]. Nowadays, the exotic swarming and assembly of CPs motivate intensive endeavors for their applications, such as variable capacitors [42], drug delivery [43,44], biological detection and repair [32,45], targeted diagnosis and therapy [46], magnetic resonance imaging [47], etc.

Among the various external fields to guide the motion of CPs, light is a powerful and versatile external stimulus and has various advantages including remote controllability and high temporospatial resolution. Thus, the light control of CPs has attracted much considerable attention. Back in 1970, Arthur Ashkin invented optical tweezers [48]. He used optical forces induced by lasers to manipulate the motion of micrometre-sized particles and neutral atoms. Hereafter, the optical tweezer technology was frequently used to trap and manipulate CPs in micro- and nanoscale [49,50,51]. In the meantime, light, acting as an energy source, was widely used to regulate the stop–go motion, speed, and direction of micro- and nanomotors because of its precisely adjustable energy input and direction [52,53,54,55,56]. Lately, vast researches have emerged on the light-controlled swarming and assembly of CPs, which promise the creation of intelligent programmable materials and reconfigurable robots [4,57] but suffer from limitations in arbitrary applicability, precise arrangement of various CPs, enrichment of applications, etc. 

Regarding the design strategies, propulsion mechanisms, motion behaviors, and emerging applications of light-driven micro- and nanomotors, we have made a detailed and tutorial review [55]. This review introduces the general principles of light-controlled swarming and assembly of CPs before amply summarizing the recent advances in the field in terms of employing optical forces, photochemical reactions, photothermal effects, and photoisomerizations. Afterwards, the potential applications, challenges, and future prospects of light-controlled swarming and assembly of CPs are also discussed. With the rapidly increasing innovations in mechanisms and strategies with easy operation, low cost, and arbitrary applicability, light-controlled swarming and assembly of CPs may offer new opportunities to develop programmable materials and reconfigurable robots for cooperative grasping, collective cargo transportation, and micro- and nanoengineering. We expect it may provide better horizons to those who wish to participate in this research field and spark the imagination of scientists.

## 2. General Principles for Light-Controlled Swarming and Assembly of Colloidal Particles (CPs)

Generally, CPs could generate autonomous motion or forced migration under a local or global chemical gradient and external fields (magnetic, electric, thermal, acoustic, flow, and optical fields). Thus, they may come together and form into swarms or assemblies through particle–particle and particle–interface interactions. Even though CP swarms and assemblies are slightly different in their structures, some general principles actually exist in their formation processes. 

**Figure 2:**
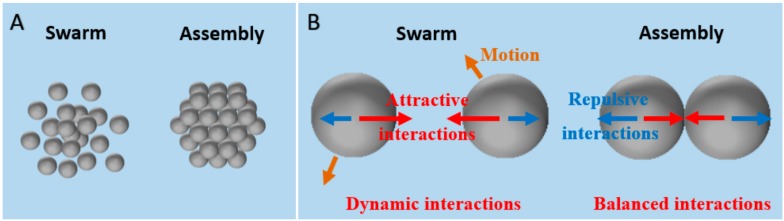
(**A**) Schematic illustration of a swarm and an assembly and (**B**) the state and mutual interactions of the individual particles in the swarm and assembly.

Swarms (herds, schools, or flocks etc.), including polar and aploar swarms [58,59], usually refer to a group of entities with autonomous motions (Figure 2A), such as microorganisms, insects, animals, or active CPs [25,60]. Individual autonomous particles in the swarm mainly interact with one another via dynamic long-range attraction and short-range repulsion (Figure 2B). The long-range attraction enables the particles to aggregate together, whereas the short-range repulsion guarantees that the particles do not collide with each other [61]. On the other hand, assemblies are hierarchical ordered structures consisting of interacting components (Figure 2A) [4]. Colloidal assemblies, such as colloidal molecules, polymers, and crystals [27,62], can only be formed if the attractive and repulsive interactions between individual CPs are sufficiently balanced in the colloidal system (Figure 2B). Hence, one of the striking features to distinguish swarms and assemblies is the state of the individual particles in the group. The individual CPs in swarms can autonomously move and are not orderly arranged, while those in (dynamic) assemblies gather in an (transient) ordered arrangement and exhibit no relative motions with respect to the assemblies (if there are no external disturbances), because of the dynamic and (transient) balanced interactions, as shown in Figure 2.

From the above analysis, one common prerequisite to realize the light-controlled swarming and assembly of CPs is to construct light-responsive short-range and long-range interactions to modulate the collective behaviors of CPs. As defined by Wang et al. [26], interactions acting on length scales smaller and comparable to the dimensions of the particles themselves are considered as short-range interactions. Otherwise, they are long-range interactions. The light-controlled short-range interactions consist of the light-controlled Van der Waals interactions (1–10 nm), steric repulsion (1–100 nm), hydrophobic attraction (1–100 nm), electrostatic interactions (1 nm–1 μm), etc. [63,64]. The light-controlled long-range interactions with an acting range from micrometers to millimeters are generally produced from direct optical forces or light–energy conversions, such as photochemical reactions, photothermal conversions, and photoisomerization, which induce diffusiophoresis, thermophoresis, convection, and Marangoni flows to regulate the collective behaviors of CPs [26,63,65,66,67]. Therefore, the photoresponses of CPs themselves or the environment (liquid media and substrates) are essential to generate the light-controlled short-range and long-range interactions to regulate the swarming and assembly of CPs. 

## 3. Light-Controlled Swarming and Assembly of CPs

Upon light irradiation, the photoactive CPs or environment (liquid media and substrates) respond to it and change their state, which could induce the swarming and assembly of CPs in the following ways. At first, the CPs are subjected to direct optical forces and gather into swarms or assemblies if a highly focused light source is applied for trapping and manipulating the CPs, namely, optical tweezers for the assembly of CPs . Secondly, photoactive CPs, liquid media, and substrates can absorb light energy to produce various photochemical reactions and photophysical effects, such as photocatalytic reactions, photolysis, photothermal conversion, photoisomerization, and so on. In this condition, gradient fields of chemicals or light-induced energies are established around the light-exposed area. Under the local gradient fields, CPs attract or repel their neighbors, thereby causing swarming and assembly of CPs or leading to the exclusion of CPs in the swarms and assemblies formed before light irradiation, respectively. With respect to the interaction nature, light-controlled swarming and assembly of CPs are classified into four categories: optical forces-maneuvered, photochemical reaction-triggered, photothermal effect-induced, and photoisomerization-controlled swarming and assembly of CPs.

### 3.1. Optical Forces-Maneuvered Swarming and Assembly of CPs

**Figure 3:**
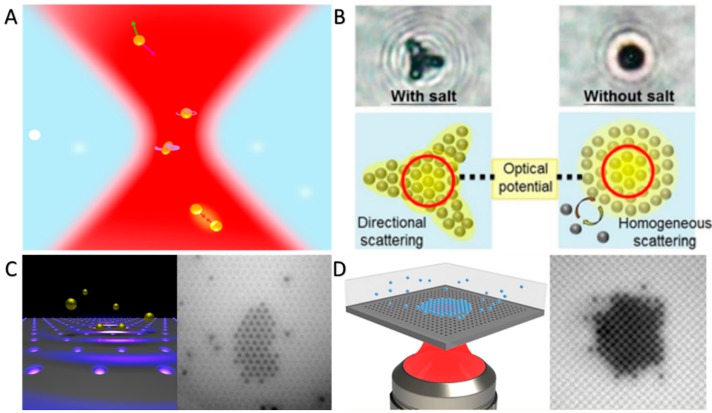
Optical forces-maneuvered assembly of colloidal particles (CPs). (**A**) Schematic illustration of manipulating metal CPs with optical forces (reproduced from [68]); (**B**) assembly of polystyrene (PS) nanoparticles induced by directional and homogeneous light scattering with or without salt (reproduced from [69]); (**C**) optical epitaxial growth of Au nanoparticle arrays (reproduced from [70]); (**D**) light-assisted and templated self-assembly of PS nanoparticles on a photonic crystal slab (reproduced from [71]).

Optical tweezers provide attractive or repulsive optical forces to precisely trap and manipulate micro- and nano-objects using highly focused laser beams. The micro- and nano-objects involve a variety of small matters, such as biological cells and a wide range of CPs. Here, we take the trapping and manipulation of colloidal metal nanoparticles as an example to illustrate the fundamental mechanism of optical tweezers (Figure 3A). Under the irradiation of incident light, the objects can absorb or scatter the photons to create momentum transfer. There are several key forces in this process, including optical gradient forces (the purple arrow) and radiation pressure (the green arrow) resulting from polarization and wavelength-dependent transfer of photon momentum [68]. Moreover, the optical forces can occur between CPs (red arrows). On the other hand, since the laser beams are the highly focused and have a high intensity, they can be used to control just one CP in an extremely tiny irradiation region. Therefore, in theory, various patterns and arrays of CPs can be realized. 

Very recently, Wang and co-workers have demonstrated the dynamic assembly of polystyrene (PS) nanoparticles (diameter: 200 nm) [69]. PS nanoparticles can be assembled into periodic structures with steady states in few minutes. It is intriguing that the scattering directions of the particles can be varied by adding salt, and thus the patterns of the assemblies are different from the situation without salt (Figure 3B). In Figure 3C, Huang et al. have showed the optical epitaxial growth of Au nanoparticle (200 nm in diameter) arrays [70]. Optical forces and optical binding make particles attach to the template, resulting in various particle arrangements. Similarly, Jaquay et al. have illustrated the light-assisted, templated self-assembly of PS nanoparticles (260 nm radius) with a photonic crystal slab. They created arrays of optical traps by a 1.55 μm laser beam, thus the PS nanoparticles could assemble in local regions and disassemble when the laser beam was turned off (Figure 3D) [71].

### 3.2. Photochemical Reaction-Triggered Swarming and Assembly of CPs

Light can trigger photochemical reactions of photocatalytic and photolytic materials, producing ions or molecules by depleting the reactants. With the diffusion of the produced ions or molecules, chemical gradient fields around CPs are established, which not only can cause CPs to perform self-propulsion [18,72], but also can induce schooling and exclusion of CPs based on diffusiophoresis. If the products of the photochemical reactions are neutral molecules, nonelectrolyte diffusiophoresis governs the motions of CPs, and the electrolyte diffusiophoresis dominates their motions if the products are ions. The velocity (*U*) of CPs near a substrate in the chemical gradient field of monovalent electrolytes under electrolyte diffusiophoresis contains two contributions, including electrophoresis (the former term) and chemophoresis (the later term), as illustrated in Equation (1) [30].

(1) $$U=\left[\frac{dln\left(C\right)}{dx}\right]\left[\frac{D_{C}-D_{A}}{D_{C}+D_{A}}\right]\left[\frac{k_{B}T}{e}\right]\left[\frac{\varepsilon （\zeta_{p}-\zeta_{w}）}{\eta }\right]+\left[\frac{dln\left(C\right)}{dx}\right]\left[\frac{2\varepsilon k_{B}{}^{2}T^{2}}{\eta }\right]\left\{ln\left[1-\tan h^{2}\left(\frac{e\zeta_{w}}{4k_{B}T}\right)\right]-ln\left[1-\tan h^{2}\left(\frac{e\zeta_{P}}{4k_{B}T}\right)\right]\right\}\;\;$$

**Figure 4:**
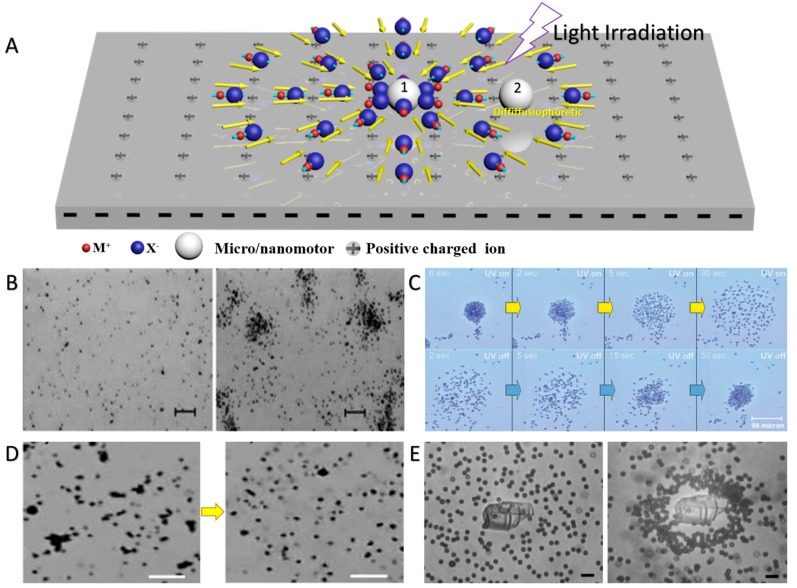
Photochemical reaction-triggered swarming of CPs. (**A**) Schematic illustration of the swarming of CPs based on diffusiophoresis; (**B**) AgCl microparticles before and after ultraviolet (UV) illumination (reproduced from [28]); (**C**) light-controlled reversible expansion–contraction movements of TiO_2_–SiO_2_ Janus microparticles (reproduced from [29]); (**D**) transition between schooling and exclusion behaviors of Ag_3_PO_4_ microparticles (reproduced from [30]); (**E**) the diffusiophoretic swarming of amino polystyrene (NH_2_-PS) CPs induced by UV light (reproduced from [74]). Scale bars, 20 μm (**B**,**D**) and 6 μm (**E**).

Here, *dln*(*C*)/*dx* is the gradient of electrolyte, *D_C_* and *D_A_* represent the diffusivities of the cations and anions, *k_B_* is the Boltzmann constant, *T* is the temperature, *e*, *ε*, and *η* represent the elementary charge, solution permittivity, and the solution dynamic viscosity, and *ζ_P_* and *ζ_W_* are the zeta potential of the particles and the substrate, respectively. Chemophoresis of CPs is usually negligible unless the M^+^ and X^−^ ions have very similar diffusivities, and electrophoresis usually dominates the electrolyte diffusiophoresis of CPs [73]. As demonstrated in Figure 4A, the M^+^ and X^−^ ions produced from the photochemical reactions diffuse away from the CPs with different rates. Then, the uneven distributed ions induce a local electric field (*E*). In return, this local *E* induces the electrophoretic propulsion (electrophoresis or electroosmosis) of the CPs inward or outward, depending on the relative magnitude of *ζ_P_* and *ζ_W_* [26], thus regulating the schooling and exclusion of CPs.

By employing photochemical reactions, various swarms have been developed based on light-triggered diffusiophoresis of CPs. Sen and co-workers reported the light-induced swarming of AgCl particles (1 μm in diameter), as demonstrated in Figure 4B [28]. In aqueous medium, the photolysis of silver chloride (AgCl) microparticles produces H^+^ and Cl^−^, as in Equation (2):

(2) $$4\mathrm{AgCl}+2H_{2}O\overset{\mathrm{hv},{\;\mathrm{Ag}}^{+}}{\to }4\mathrm{Ag}+4H^{+}+4{\mathrm{Cl}}^{-}+O_{2}$$

Because of the higher diffusivity of H^+^ ions compared to Cl^−^, an inward *E* is established, thereby triggering the inward swarming of AgCl microparticles under electrolyte diffusiophoresis, but the microparticles avoid physical contact because of the short-range repulsive electrostatic interactions between them. In addition, passive SiO_2_ particles also swarm towards the AgCl microparticles and exhibit a “predator–prey” behavior due to the long-range attractive diffusiophoretic interactions. The same group also observed a similar diffusiophoretic swarming of SiO_2_–TiO_2_ Janus particles and Ag_3_PO_4_ CPs (Figure 4C,D) [29,30]. SiO_2_–TiO_2_ Janus particles and Ag_3_PO_4_ CPs showed reversible exclusion–schooling behaviors in response to UV irradiation or ammonia addition, respectively. Furthermore, *N*-hydroxyphthalimide triflate served as a solid photoacid generator which can produce proton and triflate anion that have different diffusion coefficients, ultimately causing the light-induced diffusiophoretic swarming of passive positively charged tracers (NH_2_-PS CPs, 2 μm in diameter) (Figure 4E) [74]. There are two categories of swarms (flocks, herds, schools etc.) according to their polarity [58,59]. One category is polar swarms, in which the individual particles move with aligned velocity vectors. Examples include migrating animal herds and migrating colloidal swarms, which exhibit the displacement of the center of mass of the swarm over time [6]. Another category is apolar swarms, whose macroscopic velocity is zero even though the individual particles are polar. Examples of apolar swarms can be found in the patterns of active granular matter and living melanocytes [75,76]. It can be seen that the light-controlled swarms based on diffusiophoresis are apolar.

**Figure 5:**
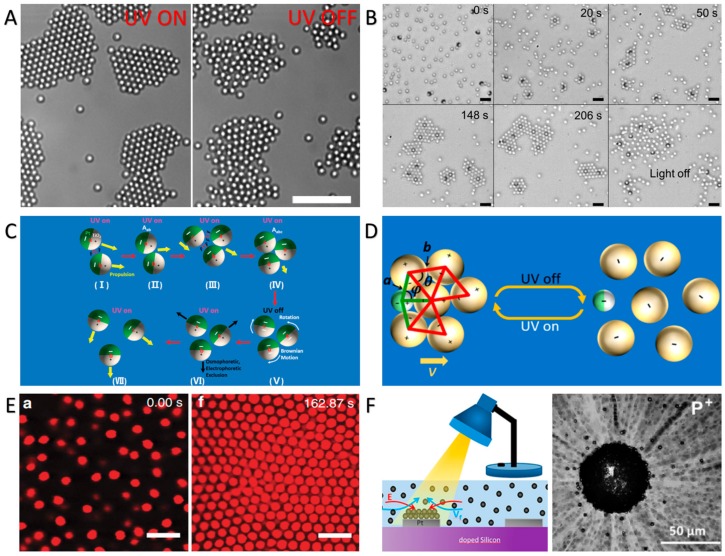
Photochemical reaction-triggered assembly of CPs. (**A**–**D**) Colloidal assembly based on photochemical reactions of CPs. (**A**) Self-assembly of bimaterial colloid surfers under blue light illumination (reproduced from [77], reprinted with permission from AAAS); (**B**) light-controlled colloidal crystal assembled by TiO_2_–SiO_2_ Janus micromotors and passive particles (reproduced from [78]); (**C**) schematic diagram of UV light-induced aggregation and separation of the TiO_2_–Pt Janus submicromotors (reproduced from [53]); (**D**) schematic diagram of light-controlled assembly and dissociation of a colloidal molecule because of the light-switchable electrostatic interactions between a TiO_2_–Pt micromotor (small green-white sphere) and SiO_2_ CPs (big yellow spheres) (reproduced from [40]); (**E**,**F**) colloidal assembly based on photochemical reactions of the substrate; (**E**) colloidal poly (methyl methacrylate) (PMMA) particles concentrating in the region of light irradiation (reproduced from [79]); (**F**) swarming of passive CPs toward the visible light-triggered micropump consisting of a Pt disk on a doped silicon wafer (reproduced from [80]). Scale bars, 10 μm (**A**); and 5 μm (**B**,**E**).

By utilizing the light-induced diffusiophoresis, light-controlled assembly of CPs can also be realized. As reported by Palacci et al., living crystals could be assembled by light-activated colloidal surfers [77]. Under blue light irradiation, such colloidal surfers photocatalytically decomposed H_2_O_2_, creating chemical gradients, and resulted in the close packing of the surfers under diffusiophoretic attractions. When the light was turned off, the assembled colloidal crystals dissociated into separated surfers because of the short-range repulsive electrostatic interactions. The assembly and dissociation of the colloidal crystals is reversible and can be swiftly controlled by regulating the light irradiation (Figure 5A). In addition, Mark et al. conducted a research on the interactions between TiO_2_–SiO_2_ Janus micromotors and passive particles [78]. Under the irradiation of UV light, TiO_2_–SiO_2_ Janus micromotors can capture and assemble passive particles into ordered structures based on diffusiophoretic effects, as illustrated in Figure 5B. Recently, we have reported that asymmetric redox reactions of H_2_O and H_2_O_2_ on the surface of TiO_2_–Pt Janus micromotors make the TiO_2_ and Pt ends oppositely charged. Thus, a swimming TiO_2_–Pt Janus micromotor under UV irradiation can capture and assemble surrounding micromotors or passive CPs on its surface, resulting in micromotor aggregates or dynamic colloidal molecules, as shown in Figure 5C,D [40,53].

Apart from the swarming and assembly based on photochemical reactions of the CPs themselves, photochemical reactions of the substrate can also induce the swarming and assembly of CPs. Solomon et al. demonstrated that a local *E* could be established around the light-exposed region of an indium–tin–oxide (ITO) glass substrate because of its photocatalytic reactions. The local *E* then induced the assembly of colloidal poly (methyl methacrylate) (PMMA) stabilized with poly(12-hydroxy-stearic acid) (PHSA) particles in the light-exposed region (Figure 5E) [79]. Complex patterns of particle assemblies could be written on the ITO glass substrate using structured light patterns. Esplandiu et al. revealed the swarming of CPs around a visible light-triggered micropump, which was simply fabricated by depositing Pt disks on a doped silicon wafer. Under light irradiation, the different photocatalytic reactions on Pt and of the silicon surface generated a gradient field of protons, resulting in an inward *E* that drove the swarming of passive CPs under electroosmosis (Figure 5F) [80].

### 3.3. Photothermal Effect-Induced Swarming and Assembly of CPs

Photothermal materials can absorb light energy and meanwhile convert it into thermal energy and induce a temperature gradient. It has also been manifested that the temperature gradient could manipulate CPs on the basis of thermophoresis. The thermophoretic velocity (*v*) of a single CP can be calculated by Equation (3) [22,67,81,82]:

(3) $$v=-D_{T}\nabla_{T}$$

**Figure 6:**
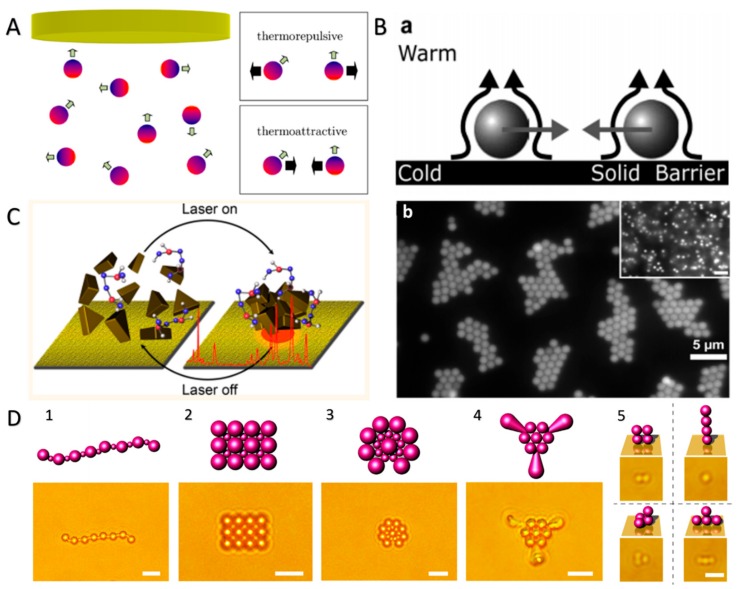
Photothermal effect-induced swarming and assembly of CPs. (**A**) Schematic demonstration of the self-propulsion and mutual interactions of metal-coated Janus spheres produced by the long-ranged temperature profiles due to light irradiation (reproduced from [81]); (**B**) CPs move ballistically to the cold surface in a temperature gradient induced by laser irradiation. The surface deflects the persisting slip flow and leads to mutual hydrodynamic attraction (a), resulting in the thermophoretic crystallization of PS CPs (b). Inset in b: the state of the particles before the presence of the temperature gradient (reproduced from [67]); (**C**) schematic diagram of the assembly and disassembly of positively charged Au nanotriangles with the laser on and off (reproduced from [83]); (**D**) 1D, 2D, and 3D hybrid assembly of 2 and 0.96 μm PS beads and anisotropic PS particles based on opto-thermophoresis; Scale bars, 5 μm (1–4) and 2 μm (5) (reproduced from [84], reprinted with permission from AAAS).

Here, ∇*_T_* is the temperature gradient across the particles, *D_T_* = *S_T_D* is the thermophoretic mobility, nd *S_T_* is Soret coefficient, and *D* is the diffusion coefficient. Figure 6A shows the thermophoretic propulsion of metal-coated Janus spheres under a local temperature gradient generated from the photothermal conversion [81]. In addition, there are different interactions between those particles depending on the sign of *S_T_*, such as thermophoretic repulsive and thermophoretic attractive interactions. Braun et al. revealed that thermophoretic interactions resulted from the thermophoretic slip flows [67]. As shown in Figure 6B, under the illumination of a laser beam, a temperature gradient arose across the particles and propelled them to the cold region (substrate). Then, a mutual hydrodynamic attraction occurred because of the presence of slip flows, thereby realizing the thermophoretic crystallization of PS CPs. Moreover, Zheng and co-workers realized a light-directed reversible assembly of plasmonic Au nanoparticles (average side length of ∼150 nm) by the thermophoretic migration of nanoparticles resulting from their photothermal effect and the associated enhanced local electric field over a plasmonic substrate (Figure 6C) [83]. Because of the high precision and highly controllability of the laser beams, the size and location of assemblies can be controlled precisely to form various patterns. The authors achieved several different dynamic manipulations of selected Au nanotriangle assemblies and demonstrated their patterns transformation. Recently, Zheng and co-workers developed a new strategy to assemble CPs, named opto-thermophoretic assembly. In this strategy, the different rates of thermophoretic migration of cetyltrimethylammonium chloride (CTAC) micelles and Cl^−^ generated a thermoelectric field. This thermoelectric field could then trap and assemble charged CPs. Figure 6D demonstrates various 1D, 2D, and 3D hybrid assemblies of various PS beads and particles based on opto-thermophoresis. The opto-thermophoretic assembly strategy releases the rigorous design rules required by the existing assembly techniques and enriches the structural complexity of the colloidal matter, which will open a new window of opportunities for basic research on matter organization, advanced material design, and applications [84].

### 3.4. Photoisomerization-Controlled Swarming and Assembly of CPs

**Figure 7:**
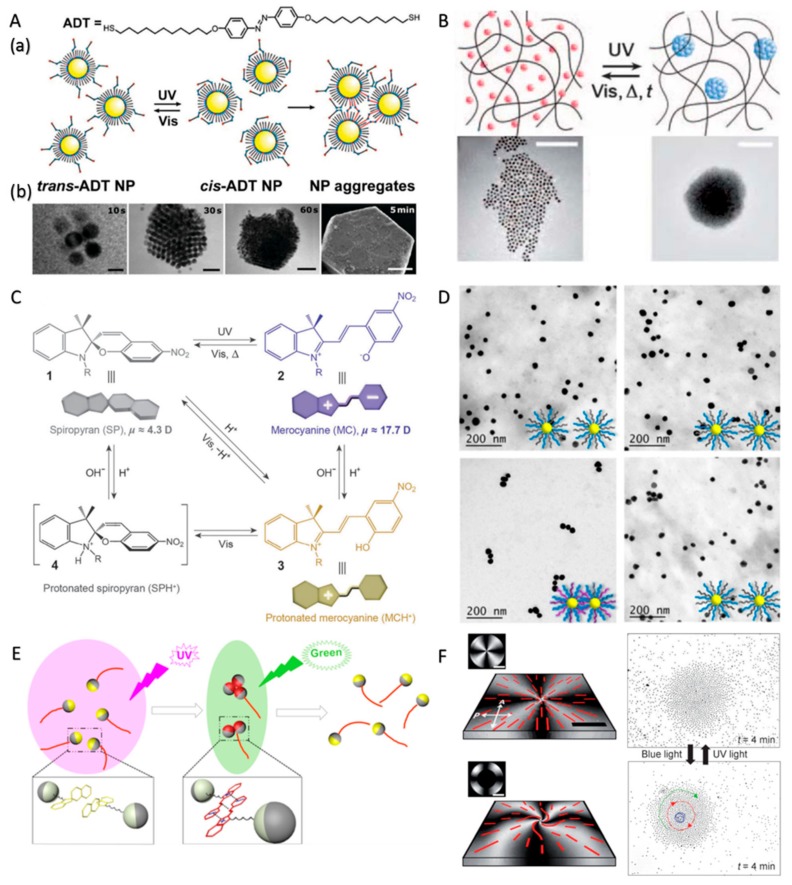
Photoisomerization-controlled assembly of CPs. (**A**) Schematic illustration of an azobenzene switch (a), assembly of nanoparticles coated with azobenzene-terminated ligands under UV light (b) (reproduced from [86]); (**B**) the dynamic aggregation of metal nanoparticles (Au or Ag) functionalized with azobenzenes under UV light, scale bar: 100 nm (reproduced from [87]); (**C**) schematic illustration of a spiropyran switch (reproduced from [88]); (**D**) the assembly and disassembly processes of amphiphilic Au nanoparticle (reproduced from [89]); (**E**) schematic diagram of light-controlled dynamic assembly of SiO_2_–Pt Janus micromotors with spiropyran moieties attached on SiO_2_ hemispheres (reproduced from [90]); (**F**) images of a cross-like and of a spiral attraction pattern formed under UV–blue light irradiation, and the corresponding particle aster and vortex after the application of an alternating current (AC) electric field (reproduced from [56]).

CPs modified with photoactive molecules, such as azobenzenes and spiropyrans, can assemble and disassemble under the control of light, during which the photoactive molecules serve as switches to change the conformations and/or properties of the CPs reversibly [4]. For example, the *trans* isomer of azobenzenes has no dipole moment, while the *cis* isomer, which is obtained under UV irradiation, has a large dipole moment (Figure 7A, (a)). Therefore, UV light can trigger dipole–dipole interactions between azobenzenes and the CPs modified with azobenzenes, inducing the light-controlled assembly of the CPs. When UV is turned off or visible-light irradiation is applied, the CPs disassemble because the dipole moment vanishes along with the *trans* isomerization of azobenzenes. By utilizing the isomerization of azobenzene-terminated ligands on nanoparticles, various metastable colloidal aggregates or crystals were obtained (Figure 7A, (b)) [4,85,86]. On the basis of the azobenzenes switches, Grzybowski et al. realized the aggregation of azobenzenes-functionalized metal nanoparticles (Au or Ag) under UV light through the *cis*–*trans* isomerization. The aggregation of the nanoparticles caused a color change of the nanoparticles because of the red shifting of the surface plasmonic resonance, allowing the writing of patterns by the structured light (Figure 7B) [87]. As for another class of photoresponsive molecule switches, the spiropyrans, as illustrated in Figure 7C, the opening of the ring gives rise to the merocyanine form and creates positive and negative charges. Thus, CPs decorated with spiropyrans can self-assemble into colloidal aggregates under UV irradiation because of short-range electrostatic interactions and then melt under visible light irradiation [88]. Chen and co-workers showed the assembly and disassembly of amphiphilic Au nanoparticles decorated with hydrophilic poly(ethylene glycol) (PEG) and hydrophobic photoresponsive polymethacrylate (PSPMA) [89]. Under the illumination of UV light, spiropyran units in PSPMA changed into the merocyanine isomer, and Au oligomers formed through π–π stacking and electrostatic attractions (Figure 7D). Similarly, Ren’s group prepared SiO_2_–Pt Janus micromotors with spiropyran moieties on the surface of the SiO_2_ hemisphere [90]. The functionalized Janus micromotors could exhibit autonomous motion taking hydrogen peroxide as fuel and meanwhile conduct dynamic self-assembly in response to light irradiation. The micromotors assembled into multimers under UV irradiation (365 nm) and melted into mono-motors immediately when UV irradiation was switched to visible light (520 nm) irradiation (Figure 7E). Simultaneously, the photoisomerization of the substrate could also induce the swarming of CPs. Sagues et al. [56] functionalized the substrate with a photosensitive self-assembled azosilane monolayer. When the pear-shaped PS microparticles were dispersed in a nematic liquid crystal confined between a photosensitive and a non-photosensitive plate, they could electrophoretically drive along the local director to form aster and vortex swarms upon application of an alternating current (AC) electric field. One of the most intriguing characteristics is that the colloidal aster and vortex could be interconverted by suitable irradiation procedures because the grafted alkyl-azobenzene chains can be reversibly switched between the *cis* and *trans* isomers (Figure 7F). The formed swarm could also be relocated to a preselected place anywhere within the experimental cell by changing the location of the UV spot.

## 4. Applications of Light-Controlled CP Swarms and Assemblies

In nature, biological swarms and assemblies can perform complex and cooperative functions. For instance, ants carry food together and wolf packs hunt a large prey cooperatively [11,12]. In analogy with the biological systems, CP swarms and assemblies have various applications related to the specific properties of individual CPs as well as collective functions attributable to the swarms and assemblies as entities, applicable for drug delivery [43,44], targeted diagnosis and therapy [46], and magnetic resonance imaging [47]. Among them, light-controlled CP swarms and assemblies have enviable advantages owing to their remotely controllability, high temporospatial precision, and non-invasive operation. Up to now, various applications of light-controlled CP swarms and assemblies have been developed.

**Figure 8:**
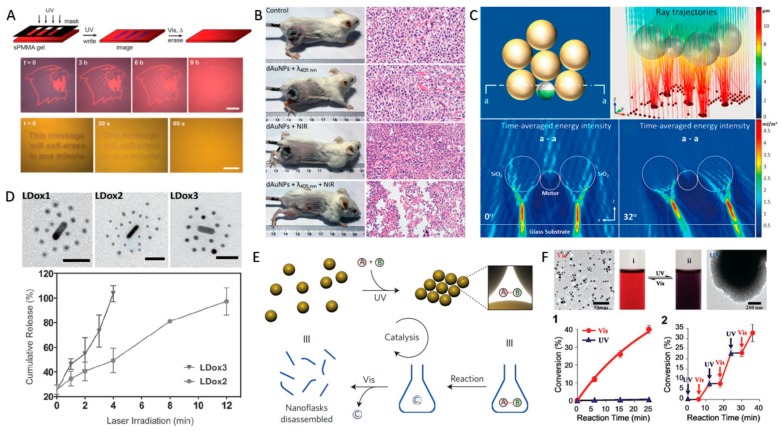
Applications of light-controlled CP swarms and assemblies. (**A**) The writing and erasing processes on an organogel “paper” containing photoresponsive Au or Ag nanoparticles inks under the control of UV light and visible light, with 0.8 s UV exposure through a transparency photomask; images obtained in Au and Ag nanoparticles films; the self-erasing of images in an Au nanoparticles film needs 9 h in daylight and 60 s in an Ag nanoparticles film by exposure to intense (0.3 m·W·cm^-2^) visible light (reproduced from [87]); (**B**) the in vivo photothermal treatment of malignant tumors by cross-linked Au nanoparticles aggregates (reproduced from [92]); (**C**) numerical simulation results illustrating that the colloidal molecule can act as a microlens array for light manipulation (reproduced from [40]); (**D**) the near-infrared (NIR) light-triggered drug release from core–satellite superstructures; scale bars are 30 nm (reproduced from [93]); (**E**) schematic demonstration of reactions in “dynamic nanoflasks” between assembled azobenzene-coated nanoparticles under UV irradiation, and the subsequent product release due to the disassembly of the cluster under visible light irradiation (reproduced from [94]); (**F**) the light-switchable catalytic activity of photoactive Au nanoparticles; Au nanoparticles decorated with photoactive ligands catalyze a hydrosilylation reaction when in dispersed state, while they have a lose catalytic activity when in aggregated state under UV irradiation (reproduced from [95]).

Firstly, the swarming and assembly can modulate the optical properties of CPs, and thus the light-controlled swarms and assemblies have promising applications in responsive optical devices and photothermal agents. For instance, Grzybowski and co-workers [87] developed an organogel “paper” containing photoresponsive Au or Ag nanoparticles inks, which were fabricated by coating Au or Ag nanoparticles with mixed self-assembled monolayers of dodecylamine (DDA) and photoswitchable azobenzene-terminated thiol. The color of the organogel “paper” experienced dynamic changes (shifting of the surface plasmon resonance) in response to UV irradiation because of the light-controlled assembly–disassembly transition of the photoresponsive Au or Ag nanoparticles. As a result, images and words could be written into the organogel “paper” by UV light, and spontaneously self-erased over time when UV was off (Figure 8A). Alternatively, Klajn et al. created self-erasing patterns in poly(ethylene glycol) gels containing spiropyrans and Au nanoparticles functionalized with 11-mercaptoundecanoic acid by utilizing the photoresponse of the medium instead of that of nanoparticles [91]. Under irradiation of visible blue light, the acidity of the medium increased and it triggered the disassembly of the nanoparticles held together by hydrogen bonds, which thus changed the color of the gel. Furthermore, Gao and co-workers [92] reported that light-triggered aggregation of Au nanoparticles could shift the surface plasmon resonance from the visible to near-infrared region, which not only enhanced photoacoustic imaging, but also improved their effectiveness in photothermal tumor ablation, as shown in Figure 8B. Very recently, we demonstrated that light-controlled TiO_2_–Pt micromotors could effectively capture and assemble spherical transparent CPs into dynamic colloidal molecules [40]. The numerical simulation results (Figure 8C) illustrated that the colloidal molecule could act as a swimming microlens array for light manipulation.

Secondly, the swarming and assembly of CPs can create inter-particle voids to load drugs or act as nanoreactors. Chan and co-workers designed DNA–nanoparticle-assembled core–satellite superstructures as carriers of therapeutic agents [57,93]. Upon near-infrared light irradiation, the loaded Dox drug was released from the superstructure because of the thermal denaturation of the DNA linkers and the disassembly of the superstructure thanks to the photothermal effect of Au nanorods, as shown in Figure 8D. In addition, Klajn et al. utilized interparticle voids in the nanoparticle assemblies as “dynamic nanoflasks” [4,94]. The “dynamic nanoflasks”, which were formed by the assembly of azobenzene-coated nanoparticles under UV irradiation, could selectively trap and concentrate small polar molecules (‘A’ and ‘B’ in Figure 8E) to enhance the rates of reactions between them. Once the products (‘C’ in Figure 8E) were generated, they could be released from the “nanoflasks” when the nanoparticle superstructure was disassembled under visible light irradiation. 

Thirdly, the swarming and assembly of CPs can regulate the surface area and the chemical activities of the CPs, thereby creating switchable or tunable catalysts. As shown in Figure 8F, Au nanoparticles covered with azobenzene units and alkyl amine ligands in dispersed state could catalyze reactions such as hydrosilylation. However, once exposed to UV light, the Au nanoparticles assembled into supraspherical aggregates, which significantly lowered the surface area of the nanoparticles and ceased the catalytic reactions. The catalytic activity was switched ‘‘on’’ and ‘‘off’’ by the light-controlled assembly and disassembly of the nanoparticles [4,95]. The aggregation of nanophotocatalysts usually reduces their photocatalytic activities because of the decreasing available surface area and active sites for photocatalytic reactions [96]. However, Sun and co-workers found that TiO_2_ nanoparticles underwent serious aggregation under UV irradiation because of the shifting of the isoelectric point of the particles, but some aggregates had a superior photocatalytic activity than the dispersed nanoparticles thanks to the facilitated charge separation and transfer between closely contacted nanoparticles [97]. 

## 5. Conclusions and Future Prospects

In conclusion, tremendous progress has been made in the light-controlled swarming and assembly of CPs by employing optical forces and light–energy conversions, including photochemical reactions, photothermal effects, and photoisomerizations. However, limitations and challenges remain. For example, optical tweezers can offer a versatile manipulation of CPs for swarming and assembly with an astonishing single particle resolution, but a highly focused light source and specific features (transparency or spherical shape, etc.) of the particles are required [98]. Photochemical reaction-triggered swarming and assembly is usually sensitive to ion concentration in the liquid medium, and photothermal effect-induced swarming and assembly require the photothermal conversion of the CPs or substrate and can only be triggered by light with a specific wavelength. Photoisomerizaion-controlled swarming and assembly are a surface chemistry-based method, posing the problem of applicability to arbitrary particle systems [99]. Furthermore, the so far reported strategies usually aim to the light-controlled swarming and assembly of CPs with the same geometrical or material features, and the precise arrangement of different CPs in a swarm or assembly is of extreme difficulty. In the future, specific attention should be paid to the development of general strategies for light manipulation of CPs regardless of their geometrical and material features. In addition, to obtain programmable materials, it is highly desired to control the precise temporospatial arrangement of different CPs in the swarms and assemblies by light. As for the application aspect, even though some proof-of-concept applications have been demonstrated, including photothermal therapy, drug delivery, pattern writing–self-erasing, switchable–tunable catalysts, etc., some important issues should be addressed before the real applications, such as safety issues in photothermal therapy and drug delivery and cost issues in others. On the other hand, the developed applications are mainly based on light-controlled assembly–disassembly of CPs. Other emergent behaviors, such as collective migrations or dynamic transformations of light-controlled swarms and assemblies, should be taken into consideration in the future. By utilizing the above-mentioned emergent behaviors, it is envisioned that, with elaborate design, CP swarms and assemblies can move to predefined working sites and transform into various user-specified microtools or microdevices, such as micrograspers, microdrillers, microwrenches, microvalves, micropumps, etc. To mimic the complex collective behaviors of biological swarms, such as cooperative carrying, migrating, foraging, nesting, and defending [11,100], it is essential to create “smart”, light-controlled, artificial swarms or assemblies comprising active CPs in response to multiple stimuli. With the rapidly increasing innovations in mechanisms and strategies with easy operation, low cost, and arbitrary applicability, light-controlled swarming and assembly of CPs may finally realize intelligent programmable materials and reconfigurable robots for cooperative grasping, collective cargo transportation, microfabrications, etc. [25,26].
